# Does Direct Benefit Transfer Improve Outcomes Among People With Tuberculosis? – A Mixed-Methods Study on the Need for a Review of the Cash Transfer Policy in India

**DOI:** 10.34172/ijhpm.2022.5784

**Published:** 2022-01-30

**Authors:** Jigna D. Dave, Mihir P. Rupani

**Affiliations:** ^1^Department of Respiratory Medicine, Government Medical College Bhavnagar, Maharaja Krishnakumarsinhji Bhavnagar University, Bhavnagar, India.; ^2^Department of Community Medicine, Government Medical College Bhavnagar, Maharaja Krishnakumarsinhji Bhavnagar University, Bhavnagar, India.; ^3^Division of Clinical Epidemiology, ICMR-National Institute of Occupational Health (NIOH), Ahmedabad, India.

**Keywords:** Cash Transfer, Financial Protection, Treatment Outcomes, National Tuberculosis Program (NTP), India, Tuberculosis Elimination

## Abstract

**Background:** A direct benefit transfer (DBT) program was launched to address the dual epidemic of under-nutrition and tuberculosis (TB) in India. We conducted this study to determine whether non-receipt of DBT was associated with unfavorable treatment outcomes among patients with TB and to explore the perspectives of patients and program functionaries regarding the program.

**Methods:** We conducted a retrospective cohort study among 426 patients with drug-sensitive pulmonary TB on treatment during January-September 2019 to determine the association between non-receipt of DBT and unfavorable treatment outcomes, which was followed by in-depth interviews of 9 patients and 8 program functionaries to explore their perspectives on challenges and suggestions regarding the DBT program. Multivariate logistic regression was applied to determine whether non-receipt of DBT was independently associated with unfavorable treatment outcomes, while the in-depth interviews were transcribed to describe them as codes and categories.

**Results:** Among the 426 patients, 9% of the patients did not receive DBT and 91% completed their treatment. Non-receipt of DBT was associated with a 5 (95% CI: 2-12) times higher odds of unfavorable treatment outcomes on multivariable analysis. Patients not owning a bank account was the primary challenge perceived by the program staff. The patients perceived the assistance under DBT to be insufficient to buy nutritious food throughout the course of treatment. The program functionaries as well as the patients suggested increasing the existing assistance under DBT along with the provision of a monthly nutritious food-kit.

**Conclusion:** DBT improved the treatment completion rates among patients with TB in our setting. Provision of a monthly nutritious food-kit with an increase in the existing assistance under DBT might further improve the treatment outcomes. Future research should determine the long-term financial sustainability for ‘DBT plus food-kit’ vs. universal cash transfers in India.

## Background

**Key Messages**

** Implications for policy makers**

- Direct benefit transfer (DBT) improves the treatment outcomes of patients with drug-sensitive pulmonary tuberculosis (TB).
- Timely disbursements of DBT and additional supplementation of nutritious food kits might further improve the treatment completion rates among patients with TB.
- The duration of DBT for patients with TB, whose treatment is extended due to a change in the treatment regimen, should be decided on a case-to-case basis.
- Innovative mechanisms for deciding the amount of DBT for the marginalized population (like migrant workers/ poorest among the poor) need to be devised for bringing about equity in the cash transfer policy.
- The long-term financial sustainability of any increase in the existing amount of DBT with the additional provision of nutritious food kits needs to be compared with universal cash transfers in India.

** Implications for the public**

Direct benefit transfer (DBT) program of credit of Indian rupees (INR) 500 (~US$ 7) per month to the bank account of patients on treatment for tuberculosis (TB) was launched in India to support their nutritional requirements. But, the benefit is not reaching 100% of the beneficiaries and among those who receive it, there is a delay in its receipt. Additionally, the assistance is not reaching the poorest among the poor due to the lack of basic documents. Patients with TB (including migrant workers) should provide the correct details of their bank account along with basic identification documents at the time of diagnosis for timely credit of DBT. The purpose of the credit is to purchase nutritious food like milk or fruits, which would help the patients to take their anti-TB medicines regularly and get cured of TB. The monetary benefit should not be used for the purchase of addictive substances.

India accounted for 26% of the global incident cases of tuberculosis (TB) worldwide in 2020.^1^ Out of the estimated global annual incidence of 5.8 million TB cases, nearly 1.5 million were reported from India in 2020, making it the country with the highest TB burden worldwide.^1^ The National Tuberculosis Program in India, launched first in 1962, has recently been renamed the National Tuberculosis Elimination Program (NTEP), moving towards its resolve of eliminating TB by the year 2025.^2^

There is a twin epidemic of TB and under-nutrition in India, with a bi-directional interaction between nutritional status and active TB.^3-5^ There is also increased mortality among patients with TB due to under-nutrition.^6-8^ To address this dual epidemic, in the year 2013, World Health Organization (WHO) released guidelines on nutritional care and support among patients with TB.^9^ India adapted these guidelines in the year 2017 with the release of country-specific ‘Guidance document: Nutritional care and support for patients with TB in India.’^10^ Assessment of nutritional status at diagnosis, nutritional counseling through a mobile application, provision of enhanced ration, and monitoring the weight gain were a few benefits enlisted in the guidance document.^10,11^ From April 1, 2018, under the nutritional support program, the Government of India launched a direct benefit transfer (DBT) scheme of Indian rupees (INR) 500 (~US$ 7) per month for all patients who are on anti-TB treatment to meet their nutritional requirements.^12^ As of now, DBT is the only scheme functional under the nutritional support program in India, however, the monetary benefit also helps in preventing catastrophic costs and improves treatment adherence.

A study conducted around the launch of the DBT program reported only 7% of patients with TB having received the monetary benefits timely, while recent evidence reports it to be 29%.^13,14^ Studies do report an improvement in treatment outcomes due to cash transfer schemes and lack of money among patients with TB as a reason for treatment interruption,^15-22^ however evidence from India provides conflicting results regarding the same.^14,23^ DBT, being a comparatively new initiative in India, it was important to understand the perspectives of NTEP program functionaries as well as patients regarding the program. With this background, we conducted this study to determine whether non-receipt of DBT under the nutritional support program was associated with unfavorable treatment outcomes among patients with TB. The study also explored the perspectives of NTEP program functionaries and that of patients with TB regarding the challenges faced and suggestions to improve the DBT program.

## Methods

###  Study Setting

The study was conducted among patients with pulmonary TB in the Bhavnagar district of Gujarat state (western part of India). Bhavnagar district has a population of 0.28 million with a literacy rate of 76%.^24^ Patients with TB are primarily diagnosed at the Designated Microscopy Centre under the Department of Respiratory Medicine and at the District TB Centre (DTC) co-located at Sir Takhtsinhji hospital of Bhavnagar. Sir Takhtsinhji hospital is the tertiary-level government hospital of Bhavnagar district. Department of Respiratory Medicine (which is a part of the hospital) diagnoses around 50%-60% of the TB cases coming to the hospital. The DTC (along with a network of decentralized government health facilities in urban as well as rural areas) caters to the rest of the 40%-50% of the patients with TB in the district (DTC is not part of the hospital). The DTC is staffed with one District TB Officer, one district program coordinator, and other laboratory and data entry operator staff. Also, for the home visit of patients with TB, the DTC in Bhavnagar is staffed with 6 TB health visitors and 2 senior treatment supervisors for the urban areas, and 8 senior treatment supervisors for the rural areas.

###  Tuberculosis Program

 Under the public sector, patients with TB in India are provided with free diagnostic as well as treatment services. The drugs are placed with someone nearby, and the patient is required to collect medicines every week (directly observed treatment). The TB health visitors pay visits to the patients’ homes for routine follow-up, eliciting adverse drug reactions and ensuring treatment completion. For drug-sensitive TB, patients are required to pay 2 follow-up visits to the clinics (at the end of the intensive phase and treatment completion).

###  Direct Benefit Transfer Program Implementation

In the year 2019, 2736 people were diagnosed with TB, and 2290 were cured in the entire Bhavnagar district in the public sector. All the data related to patients with TB is maintained in a computerized form on a nationwide online platform (NIKSHAY – https://www.nikshay.in/). To avoid cash payments, the government facilitates the DBT payments through the Public Financial Management System (PFMS – https://pfms.nic.in/NewDefaultHome.aspx). PFMS is a web-based online software to distribute various government schemes’ benefits directly to the beneficiary without any possibility of pilferage or fraud. Patients with drug-sensitive pulmonary TB are put on a daily-regimen treatment of 6 months (180 days). Under the DBT program, INR 500 (~US$ 7) per month is paid for nutritional support during the treatment duration of 6 months. The first installment, generally of INR 1000 (~US$ 14), is to be paid before the patient enters the third month of treatment; thereafter monthly payments of INR 500 (~US$ 7) till completion of treatment. However, depending on the availability of funds, this schedule is sometimes clubbed together. In case of extension of treatment, INR 1000 (~US$ 14) is paid for each extension of 2 months or if the extension is for one month, then INR 500 (~US$ 7) is paid.^12^ As soon as the patients are put on treatment, their bank account details are uploaded to NIKSHAY and the payments are made through PFMS after necessary approvals by the District TB Officer. For ease of payments, the NIKSHAY has been linked directly with the PFMS.

###  Study Design and Duration

We conducted a sequential explanatory mixed-methods research among patients with pulmonary TB in Bhavnagar. A retrospective cohort study was conducted to find out an association between non-receipt of DBT and unfavorable treatment outcomes; which was followed by in-depth interviews. The purpose of in-depth interviews of the NTEP program functionaries and the patients was to explore the challenges faced and the suggestions for improving the DBT program (constructivism paradigm and descriptive design). The theoretical framework used in the study was content analysis (systematically organizing data into a structured format).^25,26^ The quantitative component of the study was conducted from January-August 2020 and the qualitative component was conducted in September-October 2020.

###  Study Participants

*Quantitative:* Patients with drug-sensitive pulmonary TB, aged ≥18 years, notified under the public sector (and taking treatment under a government health facility) between January-September 2019 and who gave verbal informed consent to participate were included in the study. Patients who were re-treated (loss to follow-up, treatment failure, or treatment relapse) were excluded from the study.

*Qualitative:* Among the program functionaries, in-depth interviews were conducted among TB health visitors, senior treatment supervisors, the district program coordinator, and the district TB officer. Among patients with drug-sensitive pulmonary TB, in-depth interviews were conducted for those who had received their DBT.

###  Definitions (for Quantitative Component)

*Outcome variable: *‘Unfavorable treatment outcomes’ was defined when a patient was assigned a treatment outcome of loss to follow-up (stopped treatment for at least one month), or tested positive for sputum at the end of treatment (failure) or death while on treatment.^27^ ‘Successful treatment outcomes’ was defined when a patient was smear or culture negative at the end of treatment (cured) or a patient who completed treatment without evidence of failure or clinical deterioration at the end of treatment (treatment completed).^27^

*Exposure variable: * Non-receipt of DBT was defined when a patient with TB did not receive any of the installments of DBT throughout the course of treatment or even later.^12^

*Confounding variables:* The confounding variables were age, male gender, years of education, urban residence, scheduled caste/scheduled tribe caste, being single, number of family members, per-capita income, extended (vs. nuclear) family, belonging to below poverty line, currently unemployed, sputum positive TB, HIV, diabetes, asthma/Chronic obstructive pulmonary disease, having an adverse drug reaction, tobacco smoking, tobacco chewing, alcohol consumption, late receipt of the first installment of DBT and late receipt of the last installment of DBT. Late receipt of the first installment of DBT was defined when a patient with TB received the first installment [INR 1000 (~US$ 14) for the first 2 months of treatment] after the seventh day of the third month of treatment.^12^ Late receipt of the last installment was defined when a patient with TB received the last installment after treatment completion.^12^

###  Sample Size

*Quantitative: * Based on the program data obtained from the DTC of Bhavnagar, we anticipated that around 10% of the sample will not receive their DBT. Based on the findings of Sripad et al,^17^ we assumed that 9.5% of those who would receive DBT will fail to complete their treatment; whereas 26.7% of those who would not receive DBT will fail to complete their treatment. Additionally, assuming α as 5% and β as 20%, a sample size of 426 was calculated from https://sample-size.net/sample-size-proportions/ (an online sample size calculator by University of California San Francisco).^28,29^

*Qualitative: * In-depth interviews of 9 patients (saturation achieved at seventh, additional 2 interviews to confirm saturation) and 8 program functionaries (saturation achieved at sixth, additional 2 interviews to confirm saturation) were conducted. The 8 program functionaries included one district TB officer, one district program coordinator, one senior treatment supervisor, and 5 TB health visitors. None of the study participants refused participation.

###  Recruitment, Sampling, and Data Collection

*Quantitative: * A trained field investigator obtained the list of patients notified and put on treatment between January-September 2019 from NIKSHAY. This list is obtained in the form of a Microsoft Excel sheet with details like name, age, gender, address, type of residence, phone number, type of TB, and other details regarding diagnosis and treatment outcomes. From this list, 426 eligible study participants were selected for enrolment by simple random sampling.

 The field investigator filled in the basic information of the patients available from NIKSHAY; thereafter called up the patients on their phone number from NIKSHAY for confirming eligibility, verbal informed consent, and data collection. In case the phone number was invalid or wrong or unreachable, 4 attempts were made to confirm the same. In case of failure to contact the patient up to 4 attempts, the next patient on the list was enrolled in the study.

 Socio-demographic information like years of education, marital status, caste, occupation, monthly income of the family, possession of below-poverty line card, and others were obtained telephonically from the patient. Details on non-receipt, timeliness, amount and date of receipt of DBT were obtained from NIKSHAY itself (as it is linked with the PFMS). The patients were followed up passively and their treatment outcomes were also obtained from NIKSHAY.

*Qualitative: * The list of patients with drug-sensitive pulmonary TB who had received the DBT was obtained from the TB health visitors. Patients who had received the full benefit were purposefully selected (purposive sampling). The NTEP program functionaries were contacted before the commencement of the study for establishing rapport and explaining the reasons for doing the research, however, the patients were contacted on the day of the interviews. Among the NTEP program functionaries, those who were more knowledgeable, experienced, and more likely to elaborate on our questions, were purposefully selected for the in-depth interviews (purposive sampling).

 The field investigator was given an overview of the qualitative research methods by JD (first author) and was given hands-on training in conducting in-depth interviews by MR (second author). All the investigators are trained in qualitative research methods. The first author and the field investigator are females; the second author is a male. Both the authors have a Doctor of Medicine degree, while the field investigator has a degree in Master in Social Work. At the time of the study, both the authors were academic faculties in a tertiary-care medical college. However, the authors’ involvement in the research did not affect the treatment received by interviewed patients.

The interview guides were prepared by MR (second author) and were validated by an expert in the field of qualitative research (Supplementary file 1). After the informed consent procedure and consent for audio recording, the second author interviewed 3 NTEP program functionaries at his office, in which the field investigator was also present. The rest of the interviews of NTEP program functionaries were conducted by the field investigator at the office of the first author (no one else was present during these interviews). Five patients were interviewed at their home (in which TB health visitors were present), while 4 patients were interviewed on the phone – all 9 in-depth interviews of patients were conducted by the field investigator. The interviews of NTEP program functionaries took around 10-15 minutes, while the interviews of patients took around 5-7 minutes to complete. The authors neither conducted any repeat interviews nor took any field notes. Additionally, the transcripts were not returned to the participants for comment and/or correction.

###  Data Analysis

*Quantitative: * Data entry of the quantitative component was done in Epi-Info software version 7^30^ and analysis was done in IBM SPSS software version 23.^31^ The number of days to get the first and last installment of DBT, and their respective amounts were described using median and inter-quartile range (IQR). Univariable logistic regression was performed for the confounding variables like age, male gender, years of education, HIV status, and others. Those confounding variables with a *P* value of <.2 were included in the multivariable logistic regression. Multivariable logistic regression was applied using the ‘Enter’ method to determine whether non-receipt of DBT was an independent predictor of unfavorable treatment outcomes (after adjusting for confounders). Co-linearity among the variables was ruled out (tolerance values of all the variables were >0.1).

*Qualitative:* The audio recordings of the in-depth interviews were transcribed verbatim in a word document. The transcript was assigned codes manually by both the investigators together (MR and JD); any differences were reconciled through mutual agreement. The codes were grouped into categories (thematic analysis) using the Microsoft Excel software. The analysis of the assigned codes was inductive (led by our data). The analysis was not returned to the participants for their feedback.

## Results

###  Selection Process and Response Rate

**Figure 1:**
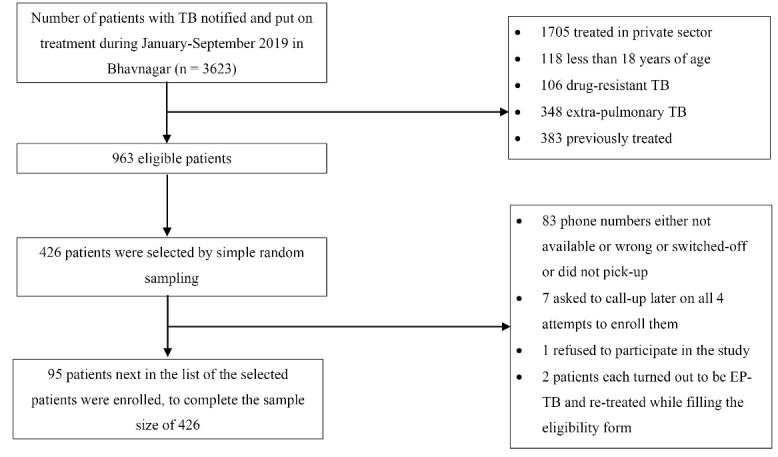
Selection Process of Patients With Drug-Sensitive Pulmonary Tuberculosis During January-September 2019 in Bhavnagar (n = 426). Abbreviations: TB, tuberculosis; EP, extrapulmonary.

Out of 426 patients enrolled in the study, we were unable to contact 83 patients (Figure 1) and 8 patients did not participate in the study (98% response rate). Additional data collection of 95 patients was conducted to complete the sample size of 426.

###  Characteristics of Patients

Among the 426 patients, the median (IQR) age was 39 (25-55) years, 68% were male, 61% had no formal education, 23% were single (vs. married), 9% belonged to scheduled caste/scheduled tribe caste and 47% were unemployed (Table 1). A majority (91%) of the patients had successful treatment outcomes, 15% of patients were tobacco smokers, 26% were tobacco chewers, 5% consumed alcohol, 4% were HIV positive and 9% had diabetes. The median (IQR) monthly family income of the patients was INR 8000 (5000-12 000) [~US$ 114 (71-171)] and 35% of the patients were living below the poverty line (defined as daily per capita expenditure less than INR 47 for urban and less than INR 32 for rural areas).

**Table 1:** Characteristics of Patients With Drug-Sensitive Pulmonary Tuberculosis During January-September 2019 in Bhavnagar (n = 426)

**Characteristics of Patients**	**Number (%) or Median (IQR)**
**Sociodemographic Characteristics**	
Age (y)	39 (25-55)
Male	289 (68)
Educational status	
No formal education	261 (61)
Primary (7th pass)	118 (28)
Secondary (10th pass) and above	47 (11)
Number of years of education	5 (0-8)
Single (vs. married)	96 (23)
Scheduled caste/scheduled tribe	38 (9)
Number of family members	5 (4-7)
Extended family (vs. nuclear family)	288 (68)
Urban residence	225 (53)
Unemployed	200 (47)
**Clinical Characteristics**	
Sputum acid-fast bacillus smear grade	
Negative	153 (36)
Scanty	37 (9)
1+	119 (28)
2+	46 (11)
3+	71 (17)
HIV positive	16 (4)
Diabetes	40 (9)
Current tobacco smoking	65 (15)
Current tobacco chewing	112 (26)
Current regular alcohol consumption	20 (5)
Current ADR of anti-TB treatment	36 (9)
Co-morbidity of asthma/COPD	17 (4)
**Treatment outcomes**	
Successful treatment outcomes	
Cured	299 (70)
Treatment completed	89 (21)
Unfavorable treatment outcomes	
Death while on treatment	23 (6)
Loss to follow up	10 (2)
Treatment failure	5 (1)
**Economic Characteristics**	
Family income in INR	8000 (5000-12 000) [~US$ 114 (71-171)]
Income of patient in INR	2000 (0-6000) [~US$ 29 (0-86)]
Below poverty line card	147 (35)

Abbreviations: IQR, inter-quartile range; INR, Indian Rupees; ADR, adverse drug reaction; COPD, chronic obstructive pulmonary disease; TB, tuberculosis.

###  Direct Benefit Transfer Parameters

Nine percent of the patients did not receive DBT, 46% received the first installment late and 49% received the last installment after their treatment completion (Table 2). The median (IQR) days after initiation of treatment to receive the first installment was 56 (33-86), while to receive the last installment was 176 (157-199). The median (IQR) days for receipt of the last installment after treatment completion was 24 (11-52). The median (IQR) amount of the first installment was INR 1000 (1000-1000) [~US$ 14 (14-14)] and the total DBT was INR 3000 (3000-3000) [~US$ 43 (43-43)].

**Table 2:** Parameters of Direct Benefit Transfer Received by Patients With Drug-Sensitive Pulmonary Tuberculosis During January-September 2019 in Bhavnagar (n = 389)

**Parameters of DBT**	**Number (%) or Median (IQR)**
Non-receipt of any DBT	37 (9*)
Late receipt of first installment of DBT	196 (50)
Number of days to get first installment of DBT from treatment initiation	56 (33-86)
Amount of DBT received in first installment in INR	1000 (1000-1000) [~US$ 14 (14-14)]
Number of days to get last installment of DBT after treatment initiation	176 (157-199)
Receipt of last installment of DBT after treatment completion	209 (54)
Number of days after treatment completion to receive the last installment of DBT (n = 209)	24 (11-52)
Total amount of DBT received in INR	3000 (3000-3000) [~US$ 43 (43-43)]

Abbreviations: IQR, inter-quartile range; INR, Indian Rupees; DBT, direct benefit transfer. * n = 426.

###  Association of Direct Benefit Transfer and Unfavorable Treatment Outcomes

On univariable logistic regression, age, years of education, single (vs. married), scheduled caste-scheduled tribe, being unemployed, HIV positive, having an adverse drug reaction, tobacco smoking, and non-receipt of DBT had a *P* value <.2 and were included in the multivariable logistic regression (Table 3). Late receipt of the first and last installment of DBT and the other confounding variables were not associated with unfavorable treatment outcomes (Supplementary file 2 – Table S1). On multivariable logistic regression, non-receipt of DBT, being unemployed and HIV positive status were significantly predicting unfavorable treatment outcomes. Non-receipt of DBT was associated with a 5 (95% confidence interval [CI]: 2-12) times higher odds, being HIV positive was associated with a 6 (95% CI: 2-23) times higher odds, and being unemployed was associated with a 4 (95% CI: 2-10) times higher odds of unfavorable treatment outcomes than their counterparts.

**Table 3:** Univariable and Multi-variable Logistic Regression for Variables Predicting Unfavorable Treatment Outcomes Among Patients With Drug-Sensitive Pulmonary Tuberculosis During January-September 2019 in Bhavnagar (n = 426)

**Variables **	**Univariable Logistic Regression**		**Multi-variable Logistic Regression**	
	**Crude OR (95% CI)**	***P*** ** Value**	**Adjusted OR (95% CI)**	***P*** ** Value**
Age (y)	1.02 (1.004-1.044)	.01	1.01 (0.98-1.03)	.71
Years of education	0.9 (0.86-1.02)	.12	0.98 (0.89-1.01)	.66
Single (vs. married)	0.3 (0.08-0.9)	.03	0.4 (0.09-1.4)	.13
Scheduled caste-Scheduled Tribe	2.1 (0.8-5.4)	.12	3 (0.9-7)	.08
Unemployed	5 (2-11)	<.001	4 (2-10)	**.001**
HIV positive	7 (2-21)	<.001	6 (2-23)	**.004**
Adverse drug reaction	2 (1-6)	.09	3 (1-8)	.06
Tobacco smoking	3 (1.2-5)	.01	2 (1-5)	.08
Non-receipt of DBT	6 (3-13)	<.001	5 (2-12)	**.001**

Abbreviations: OR, odds ratio; DBT, direct benefit transfer; CI, confidence interval.

###  Challenges and Suggestions on DBT Program Perceived by NTEP Functionaries

**Figure 2:**
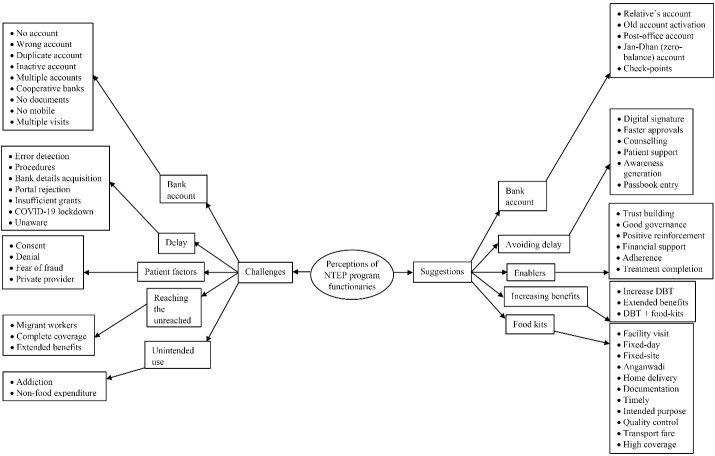
Challenges and Suggestions on DBT Program Perceived by NTEP Functionaries During September-October 2020 in Bhavnagar. Abbreviations: DBT, direct benefit transfer; COVID-19, Coronavirus disease 2019; NTEP, National Tuberculosis Elimination Program.

Among the 8 NTEP functionaries, 5 were male and their median (IQR) work experience was 13 (4.25-20) years. The challenges/suggestions on the DBT program as perceived by the NTEP functionaries was described in 5 categories: bank account, delay, patient factors/enablers, reaching the unreached/increasing benefits, and unintended use/food kits (Figure 2, refer to Supplementary file 2 – Table S2 for a detailed description of each code).

####  Bank Account

Most of the NTEP functionaries felt that not having a bank account, an inactive account, not having the required documents, and using the same bank account for multiple patients of a family were the pertinent challenges in providing DBT. Using a relative’s bank account, helping patients in opening zero-balance accounts (*Jan-Dhan yojana*https://www.pmjdy.gov.in/), and having multiple check-points while uploading bank details in the NIKSHAY portal were the suggested solutions.

“*If no one from the family has a bank account, then we send them to post-office… for TB patients, they open an account in just 10 minutes”* (TB health visitor, male, 6 years of experience).

####  Delay

 Delay in procedures, error detection, and getting correct bank details from the patients were the critical perceived challenges in providing DBT timely. The use of digital signatures of signing authority, faster approvals on the different check-points, and counseling patients to submit bank details earliest were the suggested solutions.

“*In some cases, DBT is delayed as it might be rejected by NIKSHAY or PFMS and the entire batch of beneficiaries is delayed for 15 days… the second trigger is done within 7 days to resolve it… Now all signatures are approved digitally and delay of 5 to 6 days is prevented”* (District program coordinator, female, 2 years of experience).

####  Patient Factors/Enablers

 Patients taking treatment from the private sector are generally financially stable and therefore deny the benefit of DBT. They also fear getting defrauded due to the sharing of confidential bank details. On the other hand, the majority of program functionaries felt that DBT supports patients financially, builds trust in the government sector, and also acts as positive reinforcement to complete the full course of treatment.

“*Some patients deny the assistance of 500 rupees *[~ US$ 7] *as they have some doubt or query where on and how their bank details will be used” *(Senior treatment supervisor, male, 20 years of experience).

“*Previously only 50% of patients use to complete their treatment course, but now 75% to 90% patients complete it due to DBT”* (TB health visitor, male, 20 years of experience).

####  Reaching the Unreached/Increasing Benefits

 Some of the NTEP functionaries felt that it was impossible to achieve 100% coverage of DBT due to the lack of documents among the migrant population. It was suggested to provide food kits along with DBT and to extend the benefits in case of extension of treatment duration.

“*The only problem left is that of 10%-15% patients who do not get DBT. In reality, these are the patients who require help as they do not have anything in their house, only a hut is there”* (District TB officer, male, 20 years of experience).

“*If the treatment course of 6 months is extended to 9 months, patients get the benefit of 6 months only. If treatment is extended, benefits should also be extended accordingly” *(District program coordinator, female, 2 years of experience).

“*It’s better to provide good food along with monetary benefits so that they can buy milk and dry fruits and ration kits should be provided through the program”* (District program coordinator, female, 2 years of experience).

####  Unintended Use/Food Kit

 Some program staff opined that DBT was being spent on addiction or buying things other than food. They also suggested replacing DBT with actual food kits, enabling the program to reach the unreached.

“*Benefit given under this program is mostly spent on addiction instead of buying food, particularly by male patients… there is more benefit in giving nutritious food instead of monetary benefits as the patient’s health is also improved”* (TB health visitor, female, 5 years of experience).

###  Challenges and Suggestions on DBT Program as Perceived by Patients

**Figure 3:**
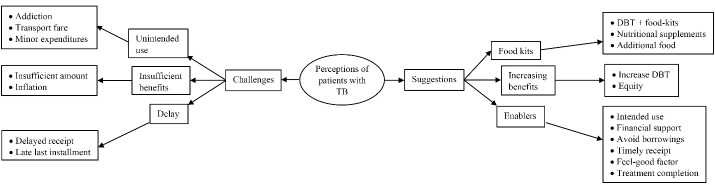
Challenges and Suggestions on DBT Program Perceived by Patients With Drug-Sensitive Pulmonary TB During September-October 2020 in Bhavnagar. Abbreviations: DBT, direct benefit transfer; TB, Tuberculosis.

Among the 9 patients, 5 were male and their median (IQR) age was 40 (20-60) years. The challenges/suggestions on the DBT program as perceived by the patients were described in 3 categories: unintended use/food kits, insufficient benefits/increasing benefits, and delay/enablers (Figure 3, refer to Supplementary file 2 – Table S3 for a detailed description of each code).

####  Unintended Use/Food Kits

 Some patients used the DBT on addiction or other minor expenditures. The majority of patients suggested providing protein powder or additional food-kits along with the DBT.

“*I use the money to buy ‘ mawa ’ *[a smokeless form of tobacco] *or fruits like apple” *(35 years male patient).

“*While the patient is on such ‘heavy’ *[high-dose]* TB drugs, it would be a big relief if some protein powder or tablets for energy or food items or drinks are provided”* (40 years female patient).

####  Insufficient Benefits/Increasing Benefits

 Almost all the patients felt that the monetary benefit under the DBT program is insufficient to meet the nutrition requirements of patients with TB on ‘heavy’ anti-TB drugs. Most of them also suggested increasing the amount under DBT and to explore the possibility of higher benefits for poorer patients.

“*Milk and fruits are required during treatment. Today, nothing much can be bought in 500 rupees *[~ US$ 7]*. My suggestion to the government is to increase this amount as 500 rupees is not sufficient. Even one liter of milk can’t be bought daily in just 500 rupees *[~ US$ 7] *a month”* (40 years female patient).

####  Delay/Enablers

 A few patients complained about the delay in receipt of the DBT installments. Almost half of the patients echoed the role of DBT in the purchase of nutritious food, treatment completion, and monetary support during the course of treatment.

“*I did not face any problem in receiving the money, but, sometimes it is delayed” *(50 years female patient).

“*See, I have completed whole TB treatment. The timely benefits helped me take my drugs regularly and now I am fine” *(35 years male patient).

## Discussion

###  Summary of Findings

 Our study determined the association of non-receipt of DBT with unfavorable treatment outcomes among patients with TB and explored the perceptions of patients and program functionaries on improving the DBT program. We found that around one in ten patients did not receive any DBT in our setting. Also, nearly half of the patients were in late receipt of their first installment of DBT, while the same percentage of patients received the last installment after completing their treatment. Despite the high treatment completion rate, patients not receiving DBT were more likely to experience unfavorable treatment outcomes. Most of the patients receiving the DBT used it for its intended purpose, however, they felt that it was insufficient to cover the nutritional costs. The NTEP program functionaries too felt that a nutritious food-kit along with the DBT would improve the treatment outcomes of the patients.

###  What Explains These Findings?

The DBT program was launched in the year 2018 and after a few teething troubles,^13^ the process of cash transfer has been easier for a while now. Even with adequate efforts by the NTEP staff, challenges like a lack of bank account explained the DBT not reaching all its beneficiaries. The late opening of bank accounts in some cases might justify the late receipt of the first installment of DBT. Also, there might be a few patients who are economically well-off and might be refusing the cash assistance provided by the government, explaining the small percentage of patients not receiving the DBT. The receipt or anticipated future payments of DBT after the start of the treatment might act as a motivation for the patients to complete their treatment. In addition, the consumption of nutritious food purchased from the DBT might have played a role in the better treatment outcomes among those receiving DBT. This might explain why those not receiving DBT failed to complete their treatment in our study.

###  Coverage and Delay of Direct Benefit Transfer

Studies assessing the DBT in the initial months of the launch of the program in 2018, reported the coverage to be as low as 29%,^13^ which increased to 53%-79%^14,23,32^ in the next few months of rollout. The present study found 91% of the patients with TB receiving DBT during the study period of January-September 2019 suggesting an improvement in the cash transfer process in due course of time. Also, the previous studies reported a median delay of 74-156 days in the receipt of the first installment,^13,14,23^ while this delay was found to be reduced to 56 days in the present study. However, the present study also found 49% of the patients receiving the last installment after treatment completion, which was reported to be 25% in a previous study.^23^ The authors believe that the low figures for the delay reported in the previous study^23^ might be due to more intense follow-up and monitoring among TB-HIV co-infected patients compared to patients with TB alone. The primary purpose of DBT is to buy nutritious food for a patient with TB, which helps them to have better compliance with the adverse drug reactions of anti-TB drugs and thereby improve their treatment outcomes.^9^ Therefore, it is imperative to avoid any delays in the processing of payments of DBT.

###  Preventing Delay of Direct Benefit Transfer

Several measures would be able to ensure timely receipt of DBT for the patients. As soon as the patient gets notified in the NIKSHAY portal, the TB health visitors should obtain the details of the bank account and the unique identification number. Under the NTEP program, TB health visitors are required to pay a home visit to the patients for identifying a drug provider nearby. Helping the patients open a bank account or zero-balance account (*Jan Dhan Yojana*) while the TB health visitors pay a visit at the patients’ homes would be the first step in avoiding any delay in uploading the bank details to the NIKSHAY portal. There is also a need to create awareness among the patients regarding the option of opening zero-balance accounts.^14^ In case a family member or relative has a bank account, a signed agreement between the family member/relative and the patient about the mutual transfer of the monetary benefit would be helpful. The next step would be early verification of the bank details by a senior treatment supervisor under the NTEP program. Once the bank details are verified, the district TB officer should give timely approvals to the list of beneficiaries so that the money can be transferred by the district program manager through PFMS at the earliest. Sufficient availability of grants needs to be ensured for timely disbursals. Finally, the beneficiaries should receive notifications on their mobile phones regarding the credit of DBT to enable its timely utilization.^14^

###  Direct Benefit Transfer and Treatment Outcomes

Cash transfer programs have been reported to be beneficial in improving treatment outcomes among patients with TB in Argentina, Brazil, Ecuador, Moldova, and Russia.^15,17-22^ Previous studies determining this association in India found conflicting results – one study reported no association, whereas another reported a significant association with 4 times more chances of unfavorable treatment outcomes among non-recipients of DBT.^14,23^ The study populations and comparison groups for both of these studies were different - the study which reported a positive association was conducted among patients with TB and the outcomes were compared within the same cohort (after adjusting for their HIV status); whereas, the study reporting no association was conducted among patients with TB co-infected with HIV and the outcomes were compared before and after implementation of the DBT scheme. Also, both these studies were conducted in the initial months of the roll-out of the DBT scheme. The present study found non-receipt of DBT being associated with 5 times higher odds of unfavorable treatment outcomes among patients with TB. Patients with TB in India have to incur costs associated with their illness, predominantly contributed by the travel cost and the loss of wages.^33,34^ The monetary benefit helps the patients overcome such costs associated with TB and also acts as an incentive to complete their treatment. We recommend continuing the DBT program in India for improving the treatment outcomes of patients with TB.

###  Secondary Results and Qualitative Findings

The current study reported HIV status and unemployment as the predictors of unfavorable treatment outcomes, corroborated by existing evidence,^35^ although with a limitation that unemployment itself is a multi-dimensional factor. We also found that around one-fourth of patients with TB consumed tobacco in some form. The prevalence of hazardous alcohol use among patients with TB was also reported to be high in our setting^36^ and there is a remote possibility of the DBT being used for such purposes. Food insecurity among households with TB patients was also stated to be high, especially among the low-income groups.^37^ This supports the need for a nutritious food-kit being distributed to the patients every month.

The findings of our in-depth interviews among NTEP program functionaries also supported the need for the distribution of food-kits, highlighting the fact that DBT is not reaching those who are not having a bank account/basic documents. They further highlight that patients belonging to a low socio-economic class are being left out of the monetary benefit (due to the lack of bank account/ basic documents), the ones who need it. Thus, the distribution of food-kits would help to reach the unreached without any delay. The qualitative analysis of perceptions of the patients also supported the program functionaries’ opinions on food-kits. The patients further felt that the existing assistance under DBT is quite less to purchase nutritious food throughout the course of treatment and suggested that an increase would further improve their compliance with the treatment. Most of our findings of qualitative research were supported by existing literature^13,14^ and seemed transferable to India as well as to other low- and middle-income countries with a high burden of TB.

###  Strengths and Limitations

Our study is one of the initial studies employing a cohort design, adjusting for important confounding variables, for determining the impact of DBT on treatment outcomes in India. However, we may not have accounted for all the possible confounders (eg, actual nutritional status) affecting the treatment outcomes. Because only 37 patients did not receive DBT (vs. 389), we did not compare the baseline characteristics among the 2 groups. The paper also adheres to the reporting guidelines for cohort and qualitative studies.^26,38^ Since some of the patients received their DBT late or after treatment completion, the temporality of association with unfavorable treatment outcomes may not have been adequately established. We calculated crude and adjusted odds ratios as the preferred effect size, instead of relative risks, for the same reason.

## Conclusion

 We conclude that patients with drug-sensitive pulmonary TB receive their DBT late, with a minor percentage not receiving at all in our setting. Providing payments of DBT improved the treatment completion rates. Timely deliveries of DBT might help in expending it on buying nutritious food during the course of treatment. Provision of a monthly nutritious food-kit with an increase in the existing assistance under DBT might further improve the treatment outcomes of the patients. Future research should determine the cost-effectiveness, long-term financial sustainability, and treatment outcomes among patients with TB for ‘DBT plus food-kit’ vs. universal cash transfers in India.

## Acknowledgements

 We thank the State Tuberculosis Cell (Government of Gujarat, India) for funding this study. We also thank the study participants and the NTEP program functionaries of the DTC of Bhavnagar for agreeing to be a part of this study. We also thank Dr. Pramod Bahulekar (MGIMS, Sewagram, Wardha) for validating the interview guides. We also thank Dr. Bhavesh Modi (State Task Force STF Chairman for NTEP Gujarat) for his support during this study. We also thank Ms. Rushita Radadiya, field investigator, for her meticulous work in data collection and entry.

## Ethical issues

 Approval was taken from the Ethics Committee of Government Medical College Bhavnagar. Verbal informed consent to participate in the study was taken from the patients on the phone. Patients were identified through their NIKSHAY IDs and the data were accessible to the research team only. Written informed consent with permission for audio recording was taken from all the participants of in-depth interviews.

## Competing interests

 Authors declare that they have no competing interests.

## Authors’ contributions

 Both the authors contributed to conception, design, definition of intellectual content, literature search, data analysis, manuscript preparation, manuscript editing, and manuscript review. The first author acquired the funding, while the second author drafted the first draft of the manuscript. Both the authors reviewed and edited the manuscript for corrections. Both the authors approve the final version of the article. Both the first authors would act as guarantors of the research.

## Funding

 State Tuberculosis Cell (Gandhinagar city, Government of Gujarat, India). The first author received a grant of Indian Rupees 156 310 for this research (grant number TB/382019/OR/26183-84/19/04-10-2019).

## Supplementary files

Supplementary file 1 contains in-depth interview guides.

Supplementary file 2 contains Tables S1-S3.
