# Supplementary material for: Does Direct Benefit Transfer Improve Outcomes Among People With Tuberculosis? – A Mixed-Methods Study on the Need for a Review of the Cash Transfer Policy in India
Source: Int J Health Policy Manag. 2022 Jan 30;11(11):2552–62. doi: 10.34172/ijhpm.2022.5784 (PMC9818107; doi:10.34172/ijhpm.2022.5784)
Supplement: Supplementary file 1 — contains in-depth interview guides. [file ijhpm-11-2552-s001.pdf]

Article title: Does Direct Benefit Transfer Improve Outcomes Among People With Tuberculosis? – A Mixed-Methods Study on the Need for a Review of the Cash Transfer Policy in India

Journal name: International Journal of Health Policy and Management (IJHPM)

Authors' information: Jigna D. Dave[1], Mihir P. Rupani[2,3]*

[1]Department of Respiratory Medicine, Government Medical College Bhavnagar, Maharaja Krishnakumarsinhji Bhavnagar University, Bhavnagar, Gujarat, India.

[2]Department of Community Medicine, Government Medical College Bhavnagar, Maharaja Krishnakumarsinhji Bhavnagar University, Bhavnagar, Gujarat, India.

[3]Division of Clinical Epidemiology, ICMR-National Institute of Occupational Health (NIOH), Meghaninagar, Ahmedabad, Gujarat, India.

(*Corresponding author: mihirrupani@gmail.com)

Supplementary file 1. In-depth interview guides

In-depth interview of tuberculosis patients receiving timely monetary benefits:

Purpose of the interview:

Good morning/evening. As has been explained to you, I am here today to know the different ways in which the monetary benefit received under the nutritional support program is being used.

Consent procedure: Verbal consent for audio recording with written informed consent for participating in the in-depth interview. Confidentiality and other points as mentioned in the consent form to be explained.

Briefing:

This is a part of a study by Government Medical College Bhavnagar among tuberculosis patients. The research question which is to be answered is "Among tuberculosis patients receiving monetary benefits under nutritional support program, for what purpose is the received money used?"

It is expected that you feel free to express your opinions. All the information will be kept confidential and anything told by you will not be linked with your names and it will not be disclosed to anyone, so feel free to give your comments and give your detailed opinion rather than just yes or no. Your suggestions can help the government to make appropriate changes in the nutritional support program. We will begin recording now.

Opening questions:

1. **Can you tell me your name and age?**
2. **How long back were you diagnosed with tuberculosis?**
3. **How much amount of money (approximately) has been received by you for the entire duration of your treatment of tuberculosis from the government?**

4. **What is your opinion on the timeliness of receipt of the monetary benefit?**
5. **What is the purpose for which government gives this monetary benefit to tuberculosis patients?** (Do not probe or give any hint for this question)

Specific questions:

1. **For what purpose did you use this money which you received from the government under the nutritional support program?**

   Probing questions (if needed):

   a. **What is your opinion on the consumption of nutritious food or nutritious supplement during the treatment of tuberculosis?**
   b. **What is your opinion on buying nutritious food or nutritious supplement with the monetary benefit which you received?**

   (**Probing questions if needed:** That's interesting, can you give me an example for what you just mentioned……?

   What do you mean by that…..?   Can you please elaborate on that……?)

2. **What is your opinion on the monetary benefit being given under this scheme?**
3. **How do you think that this scheme can be made better for the benefit of TB patients?**

Exit questions:

1. **Is there anything else you would like to say that you feel that you were not able to say during the interview?**

De-briefing:

During the interview, a few of the purposes you mentioned were………………… and a few suggestions to improve the scheme were………………………

I thank you for participating actively in this interview.

Can I call you for knowing more about this, in case I need to clarify a few of your answers?

<u>In-depth interview of NTEP program functionaries:</u>

Purpose of the interview:

Good morning/evening. As has been explained to you, I am here today to know the challenges faced in the implementation of the nutritional support program and suggestions to improve the same.

Consent procedure: Verbal consent for audio recording with written informed consent for participating in the in-depth interview. Confidentiality and other points as mentioned in the consent form to be explained.

Briefing:

This is a part of a study by Government Medical College Bhavnagar among tuberculosis patients. The research question which is to be answered is "Among RNTCP program functionaries in Bhavnagar, what are their perspectives regarding challenges faced in the implementation of the nutritional support program and suggestions to improve the nutritional support program?"

It is expected that you feel free to express your opinions. All the information will be kept confidential and anything told by you will not be linked with your names and it will not be disclosed to anyone, so feel free to give your comments and give your detailed opinion rather than just yes or no. Your suggestions can help the government to make appropriate changes in the nutritional support program. We will begin the recording now.

Can you tell your name, designation, and the number of years since you are in this position?

Opening questions:

1. **Who among TB patients is a beneficiary of nutritional support program?**
2. **What is your opinion on the importance of giving monetary benefits to tuberculosis patients?**

Specific questions:

1. **What are the challenges faced in the implementation of the nutritional support program?**

   Leading question: What is your opinion on giving actual nutritious food to tuberculosis patients instead of the current monetary benefit under this program?

   Leading question: If actual nutritious food is given to patients, what according to you can be the mechanism of delivery or implementation of such a program?

2. **What is your opinion on the timeliness of receipt of the monetary benefit by the TB patients?**

   Leading question: What are the reasons for the late receipt of the monetary benefit under the nutritional support program by patients?

Leading question: What is your opinion on many patients complaining of non-receipt of the monetary benefit under the nutritional support program?

(**Probing questions if needed:** That's interesting, can you give me an example for what you just mentioned……?

What do you mean by that…..?   Can you please elaborate on that……?)

3. **What are your suggestions to improve the nutritional support program?**

(**Probing questions if needed:** That's interesting, can you give me an example for what you just mentioned……?

What do you mean by that…..?   Can you please elaborate on that……?)

Exit questions:

1. **Is there anything else you would like to say that you feel that you were not able to say during the interview?**

De-briefing:

During the interview, a few of the challenges you mentioned were……….. and a few suggestions given by you were………….

I thank you for participating actively in this interview.

Can I call you for knowing more about this, in case I need to clarify a few of your answers?
