# Supplementary material for: Does Direct Benefit Transfer Improve Outcomes Among People With Tuberculosis? – A Mixed-Methods Study on the Need for a Review of the Cash Transfer Policy in India
Source: Int J Health Policy Manag. 2022 Jan 30;11(11):2552–62. doi: 10.34172/ijhpm.2022.5784 (PMC9818107; doi:10.34172/ijhpm.2022.5784)
Supplement: Supplementary file 2 — contains Tables S1-S3. [file ijhpm-11-2552-s002.pdf]

Article title: Does Direct Benefit Transfer Improve Outcomes Among People With Tuberculosis? – A Mixed-Methods Study on the Need for a Review of the Cash Transfer Policy in India

Journal name: International Journal of Health Policy and Management (IJHPM)

Authors' information: Jigna D. Dave[1], Mihir P. Rupani[2,3]*

[1]Department of Respiratory Medicine, Government Medical College Bhavnagar, Maharaja Krishnakumarsinhji Bhavnagar University, Bhavnagar, Gujarat, India.

[2]Department of Community Medicine, Government Medical College Bhavnagar, Maharaja Krishnakumarsinhji Bhavnagar University, Bhavnagar, Gujarat, India.

[3]Division of Clinical Epidemiology, ICMR-National Institute of Occupational Health (NIOH), Meghaninagar, Ahmedabad, Gujarat, India.

(*Corresponding author: mihirrupani@gmail.com)

Supplementary file 2. In-depth interview guides

Table S1. Uni-variable logistic regression of variables (with p-value >0.2) with unfavorable treatment outcomes among patients with drug-sensitive pulmonary TB during January-September 2019 in Bhavnagar (n=426)

Variables	Crude OR (95% CI)	p-value
Male gender	1.2 (0.6-2.5)	0.65
Urban residence (vs rural)	1.1 (0.6-2.2)	0.75
Diabetes	0.8 (0.24-2.8)	0.74
Sputum positive TB	1.2 (0.6-2.2)	0.75
Number of family members	0.9 (0.8-1.1))	0.78
Per-capita income in INR	1 (1-1)	0.35
Extended (vs nuclear family)	1.4 (0.65-2.9)	0.40
Below poverty-line	0.8 (0.4-1.6)	0.45
Asthma/COPD	2.3 (0.6-8.4)	0.21
Tobacco chewing	0.9 (0.4-1.9)	0.70
Alcohol consumption	1.9 (0.5-6.7)	0.33
Late receipt of first instalment of DBT	1.2 (0.6-2.3)	0.60
Late receipt of last instalment of DBT	0.9 (0.4-1.9)	0.78

Table S2. Perception of NTEP functionaries regarding challenges and suggestions on DBT scheme during September-October 2020 in Bhavnagar

Challenges regarding implementation of DBT perceived by NTEP functionaries		
**Categories**	**Codes**	**Description of codes**
Bank account	No account	Patient not having any bank account.
	Wrong account	Patient giving wrong bank account details.
	Duplicate account	Same bank account already registered for one family member for DBT, used for another family member – leading to rejection by NIKSHAY portal

	Inactive account	No transaction for a long time leading to deactivation of account
	Multiple accounts	One bank account registered under DBT, but patient checks passbook entry of another bank account for confirming/ withdrawing DBT credit.
	Cooperative banks	Cooperative banks not compatible with PFMS.
	No documents	Patients not having Aadhaar (Unique Identification) card (necessary for address proof)
	No mobile	Patients not having mobile (necessary for opening post-office account) for receiving one-time password
	Multiple visits	Patients have to make multiple visits to banks for opening or activating their account
Delay	Error detection	When NIKSHAY portal rejects the bank account number after 10-15 days, the NTEP staff suspect an error in the bank account details provided.
	Procedures	The bank account is verified at multiple levels – first by health visitor, then by district program coordinator and then goes in final approval by district TB officer (procedure of one patient takes around 10-15 days to complete).
	Bank details acquisition	If acquisition of bank details from patient gets delayed, then overall credit of DBT gets delayed.
	Portal rejection	NIKSHAY or PFMS portal may reject the bank account due to any reason and then the entire batch is delayed by 15 days. Second trigger is done within 7 days to resolve it.
	Insufficient grants	DBT getting delayed due to lack of availability of funds for disbursement.
	Covid-19 lockdown	Lockdown during Covid-19 delayed approvals of DBT.
	Unaware	Lack of awareness among patients regarding submitting bank details as soon as diagnosed with TB.
Patient factors	Consent	Patient as well as relative's consent to be taken when patient submits relative's bank account for DBT credit.
	Denial	Economically well-off patients deny the DBT assistance.
	Fear of fraud	Patient fearing the possibility of fraud by sharing bank account and Aadhaar (Unique Identification) details
	Private provider	Patients taking treatment from private providers are left out many a times due to lack of sharing of details with the NTEP staff by private doctors.
Reaching the unreached	Migrant workers	Migrant population having address proof of their native state or not owning a bank account
	Complete coverage	Inability to give the DBT benefit to 100% patients with TB due to lack of bank account/ documents
	Extended benefits	If the course of treatment is extended, the DBT benefits are not extended.
Unintended use	Addiction	DBT spent on tobacco or alcohol
	Non-food expenditure	DBT spent on non-food household expenditures
**Suggestions on improving DBT scheme perceived by NTEP functionaries**		
**Categories**	**Codes**	**Description of codes**
Bank account	Relative's account	Using relative's account in case the patient or his family members do not have a bank account
	Old account activation	Activating deactivated account with the help of health visitors

	Post-office account	Opening a post-office account for the patient
	Jan-Dhan (zero balance) account	Opening zero-balance accounts in banks under the Jan-Dhan Yojana (https://www.pmjdy.gov.in/)
	Check-points	Verifying the bank details at multiple check-points (health visitor, district program coordinator, final approval by district TB officer)
Avoiding delay	Digital signature	Digital signature by the district TB officer helps in expediting the process
	Faster approvals	Avoiding any administrative delays at the level of NTEP staff
	Counselling	Counselling patients for providing bank details earlier. Also, counselling any patient of private provider who is denying DBT.
	Patient support	Earlier provision of bank details and other documents by patients
	Awareness generation	Generating awareness among patients regarding the DBT benefits and its purpose.
	Passbook entry	Asking patient to update their passbook for entries of recent transactions would make them aware about the DBT credit
Enablers	Trust building	DBT builds trust of patients on the government sector
	Good governance	Government proactively made attempts to make the system of DBT transfer faster and smoother
	Positive reinforcement	DBT acts as a motivation for patients while on treatment for TB
	Financial support	DBT acts as a financial support for patients in the low socioeconomic class
	Adherence	Nutritious food consumption purchased from DBT every month helps overcome the adverse drug reactions and increases compliance to the treatment
	Treatment completion	The idea of getting DBT every month till treatment completion helps patients to actually complete their treatment.
Increasing benefits	Increase DBT	Increasing the amount of DBT would benefit the patients for purchase of sufficient nutritious food for the entire duration of treatment
	Extended benefits	In case the treatment of patient is extended due to any reason (most commonly patient put on drug-resistant regimen from drug-sensitive), the DBT benefits also needs to be extended till the patient is on anti-tuberculosis drugs.
	DBT + food-kits	Food kits should be given along with the DBT
Food-kit	Facility visit	Food kits can be distributed from the nearest public health facilities
	Fixed-day	Food kits can be distributed on a fixed day
	Fixed-site	Food kits can be distributed from a pre-decided and permanent site where the patients can visit for collection
	Anganwadi*	Food kits can be distributed through Anganwadi.
	Home delivery	Food kits can be delivered at home of patients by health visitors during their monthly visit to the patients.
	Documentation	Food kit distribution can be documented with photographs.
	Timely	Food kit can be distributed as soon as the patient is diagnosed with TB.
	Intended purpose	Food kit will directly serve the intended purpose of current DBT program

	Quality control	A quality control mechanism can be in place, if and when, the food-kit distribution program is rolled out.
	Transport fare	Transport fares can be reimbursed to the patients in case they travel to far-away public health facilities for collection of food-kits.
	High coverage	100% coverage of food-kit distribution can be achieved

* Anganwadi (https://wcd.nic.in/schemes/anganwadi-services-scheme) are government-run centers (mostly located in urban slums and villages) for every 800-1000 population, where nutritional supplements are given to children, pregnant women, and lactating women.

Table S3. Perception of patients with TB regarding challenges and suggestions on DBT scheme during September-October 2020 in Bhavnagar

**Challenges regarding DBT scheme perceived by patients with TB**		
**Categories**	**Codes**	**Description of codes**
Unintended use	Addiction	Use of DBT for purchase of tobacco or alcohol
	Transport fare	Use of DBT for covering costs of travel
	Minor expenditures	Use of DBT for non-food personal expenditures
Insufficient benefits	Insufficient amount	DBT amount perceived to be insufficient to purchase nutritious food throughout the duration of treatment
	Inflation	Amount of DBT not according to the rising inflation.
Delay	Delayed receipt	Delay in receipt of DBT
	Late last installment	Last installment of DBT delayed
**Suggestions on improving DBT scheme perceived by patients with TB**		
**Categories**	**Codes**	**Description of codes**
Food kits	DBT + food kits	Provision of food-kits along with DBT perceived as more beneficial by the patients.
	Nutritional supplements	Need for nutritional supplements like protein powder or energy drinks during the course of treatment of TB
	Additional food	Need for additional food/ ration, apart from that purchased from the amount received through DBT
Increasing benefits	Increase DBT	Increase the amount of DBT, in order to purchase nutritious food throughout the duration of treatment
	Equity	Patients belonging to low socioeconomic class should get higher DBT amount
Enablers	Intended use	Patients using DBT for purchase of nutritious food like milk, vegetables, fruits, etc.
	Financial support	DBT acting as a financial support for patients with TB
	Avoid borrowings	DBT avoids borrowing money from anyone as patients are able to buy food from the DBT
	Timely receipt	DBT is received timely
	Feel-good factor	Patients feel happy that some amount of money is being credited to their bank account
	Treatment completion	DBT acts as a motivation for patients to complete their treatment of six months
